# Expression of Kisspeptins and Kiss Receptors Suggests a Large Range of Functions for Kisspeptin Systems in the Brain of the European Sea Bass

**DOI:** 10.1371/journal.pone.0070177

**Published:** 2013-07-23

**Authors:** Sebastián Escobar, Arianna Servili, Felipe Espigares, Marie-Madeleine Gueguen, Isabel Brocal, Alicia Felip, Ana Gómez, Manuel Carrillo, Silvia Zanuy, Olivier Kah

**Affiliations:** 1 Instituto de Acuicultura de Torre de la Sal, CSIC, Torre de la Sal, s/n, Ribera de Cabanes, Castellón, Spain; 2 Research Institute in Health, Environment and Occupation, INSERM U1085, Université de Rennes 1, Campus de Beaulieu, Rennes, France; University of Cordoba, Spain

## Abstract

This study, conducted in the brain of a perciform fish, the European sea bass, aimed at raising antibodies against the precursor of the kisspeptins in order to map the kiss systems and to correlate the expression of kisspeptins, kiss1 and kiss2, with that of kisspeptin receptors (kiss-R1 and kiss-R2). Specific antibodies could be raised against the preprokiss2, but not the preoprokiss1. The data indicate that kiss2 neurons are mainly located in the hypothalamus and project widely to the subpallium and pallium, the preoptic region, the thalamus, the pretectal area, the optic tectum, the torus semicircularis, the mediobasal medial and caudal hypothalamus, and the neurohypophysis. These results were compared to the expression of *kiss-R1* and *kiss-R2* messengers, indicating a very good correlation between the wide distribution of Kiss2-positive fibers and that of *kiss-R2* expressing cells. The expression of *kiss-R1* messengers was more limited to the habenula, the ventral telencephalon and the proximal pars distalis of the pituitary. Attempts to characterize the phenotype of the numerous cells expressing *kiss-R2* showed that neurons expressing tyrosine hydroxylase, neuropeptide Y and neuronal nitric oxide synthase are targets for kisspeptins, while GnRH1 neurons did not appear to express *kiss-R1* or *kiss-R2* messengers. In addition, a striking result was that all somatostatin-positive neurons expressed-*kissR2*. These data show that kisspeptins are likely to regulate a wide range of neuronal systems in the brain of teleosts.

## Introduction

Reproductive activities are tightly controlled by neuronal networks, in which the GnRH system plays a central role. In turn, the activity of GnRH neurons is modulated by sexual steroids, the environment and by a series of nutritional parameters. While it has been postulated for many years that these factors act directly onto the GnRH-expressing cells, a wealth of data obtained over the last 10 years now suggest that neurons expressing kissspeptins are in fact key players in controlling the cyclic activity of the reproductive axis, possibly by activating GnRH neurons at the time of puberty [1]–[3]. However, these studies have been conducted mostly in mammals, particularly in rodents, so that very little is known on the significance of kisspeptin systems in controlling reproductive related events in other vertebrates.

In mammals, kisspeptins are derived from a single gene named *KiSS-1*, which generates a large precursor that is subsequently cleaved in several peptides among which kisspeptin 1–10 is the shortest biologically active one. However, according to phylogenetical studies, an ancestral *kiss* gene diversified through gene duplication and gene deletion in the different classes of vertebrates. Accordingly, the number of *kiss* genes varies among vertebrates from none in birds to three in Xenopus. Until now, only one *kiss* gene has been reported in lepidosaurians, while most teleost fishes have two genes, named *kiss1* and *kiss2*. Interestingly, up to date there is no evidence for the presence of any *kiss* gene in birds [4]–[7]. The same holds true for the kisspeptin receptor. While there is a single receptor in mammals (KISS1R or GPR54), the number of kiss receptors is also subject to change among vertebrate groups according to duplication/deletion. In the current stage of our knowledge, most teleost fish would have two kisspeptin receptors named *kiss-R1* and *kiss-R2* [4], [6], [7].

If it seems clear that kisspeptins are important for the control of reproduction in mammalian models [1], the situation is far from being solved in teleost fishes which, because of their long evolutive history and their important diversity, always exhibit some level of interspecies variations. Functional studies published until now suggest either a stimulatory [8], [9] or a negative effect of kisspeptin on gonadotrophin release [10]. Other studies have intended to monitor changes in kisspeptin or kisspeptin receptor expression in the brain of several species according to the sexual cycle [11]–[15]. However, in the absence of accurate information regarding the sites of expression of either the ligand or the receptor, it is quite difficult to properly interpret those data.

Until now, the distribution of kisspeptins has been documented in the brain of fish mainly on the bases of in situ hybridization [16]–[20]. Unfortunately this technique does not provide information on the projections of the kiss mRNA expressing cells, which are documented only in the zebrafish [18]. These studies showed some interspecies differences in the sites of expression of *kiss1* and *kiss2* [16]–[20]. In the European sea bass, *kiss1*-expressing cells were consistently detected in the habenula and, in mature males and females, in the rostral mediobasal hypothalamus. In both sexes, *kiss2*-expressing cells were consistently detected at the level of the preoptic area, but the main *kiss2* mRNA-positive population was observed in the dorsal hypothalamus, mainly above, but also below, the lateral recess of the third ventricle [20].

The present study aimed at providing more accurate information on the organization of Kiss/Kiss-R systems in the European sea bass (*Dicentrarchus labrax*), a perciform fish of high commercial interest. The sea bass is an interesting model given that a brain atlas is available for this species [21]–[23], its reproductive physiology is well documented [24], [25] and its GnRH systems have been investigated in great details [26]–[28].

Consequently, a first aim of this study was to raise antibodies against the Kiss1 and Kiss2 precursors using the same strategy that proved successful in zebrafish [18]. A second objective was to map the kiss systems and to correlate the expression of kisspeptins, Kiss1 and Kiss2, with that of kisspeptin receptors (*kiss-R1* and *kiss-R2*) in the brain of the European sea bass. Other objectives were to address the potential expression of *kiss-R* in GnRH1 neurons and to try characterizing the phenotype of the numerous cells expressing *kiss-R*.

## Materials and Methods

### Animals and Tissue Collection

Animals were sacrificed in accordance with the Spanish (Royal Decree Act 53/2013) and European (2010/63EU) legislations concerning the protection of animals used for experimentation. The protocol used to sacrifice the animals was approved by the Welfare Committee of the IATS (Number of Register 09-0201), supervised by the Ministry of Rural and Marine Environment. All steps were taken to reduce suffering of the animals.

Male and female European sea bass (*Dicentrarchus labrax*) were reared and maintained in the facilities of the Instituto de Acuicultura de Torre de la Sal (Spain, 40° NL) under natural photoperiod and temperature. For *in situ* hybridization (ISH) and immunohistochemical (IHC) studies, adult females and males of 3 and 2 years of age (n = 4–6 per sex) respectively, were sampled during their first sexual maturation including the non breeding (September) and the breeding season (February). The sexual maturation classification was as reported previously [29], [30].

Animals were anesthetised in 0.1% phenoxyethanol (Merck Schuchardt OHG, Hohenbrunn, Germany) and then perfused through the heart using a peristaltic pump with 50 ml of 0.65% NaCl and a fixative solution (4% paraformaldehyde, 0.1M phosphate buffer, pH 7.4, and 5% picric acid picric). Tissues were collected, fixed overnight at room temperature, dehydrated, embedded in paraffin and cut transversally in series at 6 µm. All sections were mounted onto poly-L-Lysine-coated slides and keep at 4°C.

### Generation of Specific Antibodies Against Kiss1 and Kiss2 Peptides (Table 1)

Polyclonal antibodies against the sea bass N-terminus of kiss1 and C-terminus of Kiss2 precursor sequences were raised in rabbit by GenScript USA Inc (Piscataway, NJ, USA). Both Kiss1 and Kiss2-derived peptides were chemically synthesized, purified and finally certificated by HPLC. The synthetic peptide QDVSSYN corresponding to the preprokiss1 residues 87–92 and ELEVPT corresponding to the preprokiss2 residues Kiss2 117–122 were strategically chosen as antigens in order to avoid cross-reactivity. The purified and conjugated immunogenic peptides were injected to two rabbits according to manufactureŕs protocol. After the third and final immunizations antisera were collected and purified by affinity column. Lastly, purified Kiss1 and Kiss2 antibodies were validated by ELISA.

### Analysis of Antibody Specificity

The specificity of both Kiss1 and Kiss2 antibodies used in the present study was thoroughly assessed. First, a dot-blot immunoassay was performed according to [31] with minor modifications. Briefly, 10–12 µg of synthetic Kiss1-7 peptide (QDVSSYN), Kiss1-15 (QDVSSYNLNSFGLRY) and kiss2 (ELEVPT) were immobilized in duplicate on a PVFD membrane (Immun-blot 0.2 µM, Bio-Rad laboratories Inc.CA, USA) using a Bio-dot microfiltration apparatus (Bio-Rad laboratories Inc.CA, USA). The membrane was saturated for 1 hour in Tris buffer saline (pH 7.4 with 0.05% Triton: TBST) containing 5% of nonfat dry milk and incubated with kiss1 and kiss2 antibodies diluted in 5% milk containing-TBS (1∶1000 and 1∶2000) respectively for 1.5 hour at room temperature. The membranes were then washed in TBST (2 X 10 min) and the slices incubated for 1 hour in horseradish peroxidase-conjugated goat anti-rabbit IgG at a 1∶1000 dilution (GAR-HRP, Invitrogen Molecular Probes, Eugene OR, USA). Finally, the membrane was revealed for 1 minute by chemiluminescence using Pierce ECL Western Blotting substrate (Pierce, Thermo Scientific) and detected using a Versa Doc Imaging System 5000 (Bio-Rad laboratories Inc.CA, USA).

An immunohistochemical control was carried out in Chinese hamster ovary (CHO) cells in order to detect the native form of Kiss1 and Kiss2 prepropeptides. Cells were grown in DMEM (Gibco-BRL, Eggenstein, Germany) supplemented with 5% fetal bovine serum (FBS) and distributed (in antibiotic free medium) in 1.9 cm^2^ wells containing sterile glass coverslips until 50% confluence was reached. Cells were next transfected in duplicate using FuGENE® HD transfection reagent (Promega, Madison, WI). Expression plasmids containing the full cDNAs of kiss1 or kiss2 inserted in pcDNA3 (Invitrogen) (pcDNA3-Kiss1 and pcDNA3-Kiss2) were used for transfection. The pEGFP-N3 plasmid (Clontech) was co-transfected in each case to control transfection efficiency. The cells were incubated for 36 hours at 37°C, washed with PBS and fixed in 4% paraformaldehyde. Before the immunodetection, cells were washed with PBS (2×10 min) and then blocked for 1 hour PBS-FBS (5%) at room temperature. Samples were washed with PBST (2×10 min) and then incubated with Kiss1 or Kiss2 antibody for 1.5 hour and diluted in PBS-FBS at 1∶1000 and 1∶2000, respectively. The primary antibodies were removed by washing with PBST (3×10 min) and all sections were incubated for 1.5 hour with Alexa 594 Goat anti rabbit (Invitrogen Molecular Probes, Eugene OR, USA Molecular Probes, Eugene OR, USA) diluted 1∶2000 (except in the negative control for the secondary antibody background signal). Finally, slides were mounted with Vectashield containing DAPI (Vector laboratories).

### PreproKiss2 Immunohistochemistry

For immunohistochemistry, the sections were deparaffinized in xylene (2×10 min) at room temperature and dehydrated through decreasing concentrations of ethanol. Sections were washed twice in 0.1 M phosphate-buffered saline (PBS) and then were transferred to staining dish for antigen retrieval (Tris-HCl 50 mM, pH 9.5; 1 hour at 80°C). The slides were allowed to cool down for 20 minutes before being washed twice in PBST (0.1% Triton) and blocked in PBS-BSA 1% for 45 minutes at room temperature. The samples were incubated overnight in a humid chamber at room temperature with the antibodies to preproKiss2 (1∶2000) in PBS-BSA (1%). The sections were then washed in PBST (2×10 min) and incubated for 1.5 hours (in a dark chamber at room temperature) with Alexa 594 labelled goat anti-rabbit secondary antibodies (Invitrogen Molecular Probes, Eugene OR, USA) diluted 1∶300 in PBS-BSA (1%).

### Molecular Cloning of Two Kisspeptin Receptor Genes and Riboprobes Synthesis

The probes for sea bass *kiss-R1 (accession number JN202446) and kiss-R2* (*accession number JN202447*) were synthesized using pGEM-T easy vector (Promega, Madison, WI). Antisense and sense mRNA probes were obtained with DIG RNA labeling MIX (Roche Diagnostic, Indianapolis, IN) by *in vitro* transcription with SP6 and T7 RNAs polymerases (Promega, Madison, WI) and linearized with *SacI*, *SacII* and *SalI*. Information on plasmids and restriction enzymes information are detailed in Table 2. To confirm the specificity of the *kiss-R1* and *kiss-R2* probes, parallel series of slides were always hybridized with the correspondent sense RNA probes. This procedure yielded no signal (data not shown).

**Table 2:** Detailed information on the plasmids and restriction enzymes.

Gene	Plasmid	Size (pb)	GenBank Accession number	Enzyme/RNA pol
*kiss-R1*	PGEM-T easy	1,285	JN202446	Antisense:*SacII*/Sp6 Sense:*SacI*/T7
*Kiss-R2*	PGEM-T easy	2,360	JN202447	Antisense: *SalI*/Sp6 Sense:*SacII*/Sp6

### Kiss-R1 and kiss-R2 in situ Hybridization

The in situ hybridization protocol was performed according to Escobar et al. (2013). Before the hybridization procedure, all slides were dewaxed in Ottix plus (Diapath, Italy; twice for 10 minutes) at room temperature and dehydrated through decreasing concentrations of ethanol. Sections were washed twice in 0.1 M phosphate-buffered saline (PBS), pH 7.4, followed by refixation in 4% paraformaldehyde diluted in PBS for 20 minutes. After washing (PBS), sections were incubated in proteinase K for 5 minutes at room temperature (10 mg/ml in 50 mM Tris-HCl, pH 8.0, 5 mM EDTA), rinsed and post-fixed in 4% paraformaldehyde. Sections were rinsed twice in saline-sodium citrate (SSC) 2X at room temperature. Hybridization was performed overnight at 65°C in a humidified chamber using 100 µl of hybridization buffer (2X SSC; 2.5% dextran sulfate; 50% deionized formamide; 5 X Denhardt’s solution; 50 µg/ml of yeast tRNA, pH 8.0; 4 mM EDTA) containing the DIG-labeled kiss-R1 and kiss-R2 probes (1.5 µg/ml). On the next day, slides were rinsed in 2X SSC at 65°C, followed by two rinses at 65°C (2×30 minutes) in 2X SSC/50% formamide. Final rinses were made in 0.2 and 0.1X SSC at room temperature and sections were processed for immunodetection. The sections were washed for 10 minutes in 100 mM Tris-HCl buffer, 150 mM NaCl, pH 7.5, and then incubated for 30 minutes in the same buffer containing 0.5% blocking reagent and 0.2% Triton X-100. They were then incubated overnight at room temperature in alkaline phosphatase-conjugated sheep Fab fragment antibodies to digoxygenine (Roche Diagnostic, Indianapolis, IN) diluted to 1∶2000. The next day, sections were incubated for fluorescent detection with HNPP (2-hydroxy-3-naphtoic acid-2′-phenylanilide phosphate) in HNPP/FastRED solution at room temperature (Roche Diagnostic, Indianapolis, IN) for 3 hours.

### Combined Kiss2 mRNA in situ Hybridization and Immunohistochemical Detection of Kiss2

To corroborate the specificity of the antiserum to preproKiss2, a combination of in situ hybridization and immunohistochemistry was performed on the same sections. *Kiss2* mRNAs expression sites were detected by ISH as described above. The sections were then rinsed twice in PBST (10 min) and immersed in Tris-HCl 50 mM buffer (pH 9.5) for 1 hour at 80°C for antigen retrieval, washed twice in PBST (10 min) and subsequently blocked for 45 minutes in 0.2% Triton PBS (containing 0.5% dry fat milk) at room temperature before being exposed overnight to the primary antibody (1/2000). On the next day, sections were washed three times in 0.2% Triton PBS and subsequently incubated with Alexa 488 goat anti-rabbit (Invitrogen Molecular Probes, Eugene OR, USA) for 2 h at room temperature.

### Combined Kiss-R2 mRNA in situ Hybridization and Immunohistochemical Detection of GnRH1, TH, nNOS, NPY and SRIF

In order to identify the phenotypes of *kiss-R2* mRNA-expressing cells in delimited brain areas, a double labeling protocol was performed by combining *in situ* hybridization and immunohistochemistry (Table 1).

**Table 1:** Detailed information on the antibodies.

Protein	Species	Antigen	References	Dilution
Kiss2	Rabbit	ELEVPT	This study	1∶1000
Seabream GAP (sbGAP)	Guinea pig	Sea bass sbGAP	Gonzalez-Martinez et al., 2002	1∶200
Neuronal NO-synthase (nNOS)	Rabbit	Pat nNOS	Herbison et al., 1996	1∶1000
Neuropeptide Y (NPY)	Guinea pig	NPY	Ciofi et al., 1987	1∶5000
Tyrosine Hydorxylase (TH)	Rabbit	Bovine pheochromocytoma	Thibault et al., 1981	1∶1000
Somatastatin (SRIF)	Rabbit	SRIF 1–14	Dubois et al., 1975	1∶1000

Once *in situ* hybridization was revealed, the sections were rinsed twice in PBST and immersed in Tris-HCl buffer (50 mM pH = 8) antigen retrieval four 1 hour at 80°C, washed twice in PBST (10 min) and subsequently blocked for 45 minutes in 0.2% Triton PBS (containing 0.5% dry fat milk) at room temperature and incubated over night at room temperature using: Anti sbGAP (1∶200) previously characterized and validated [32], rat nNOS (1∶1000) [33], [34], neuropeptide Y (1∶5000) [35], tyrosine hydroxylase (1∶1000) [36] and somatostatin 1–14 (1∶1000) [37], [38] (Table 1). The next day, sections were washed three times in 0.2% Triton PBS and subsequently incubated with Alexa 488 goat anti-guinea pig (Invitrogen Molecular Probes, Eugene OR, USA) for 2 h at room temperature.

All slides were washed in PBST (2×10 min) and finally coversliped and mounted in Vectashield containing DAPI (Vector Laboratories) and observed in an epifluorescence microscope (Olympus Provis). The images were processed with the Olympus Analysis Cell B software and figures illustrated with Photoshop 7.0. The nomenclature for brain nuclei is taken from the sea bass atlas [21]–[23].

## Results

### Antibodies Against European Sea Bass Kiss2

**Figure 1:**
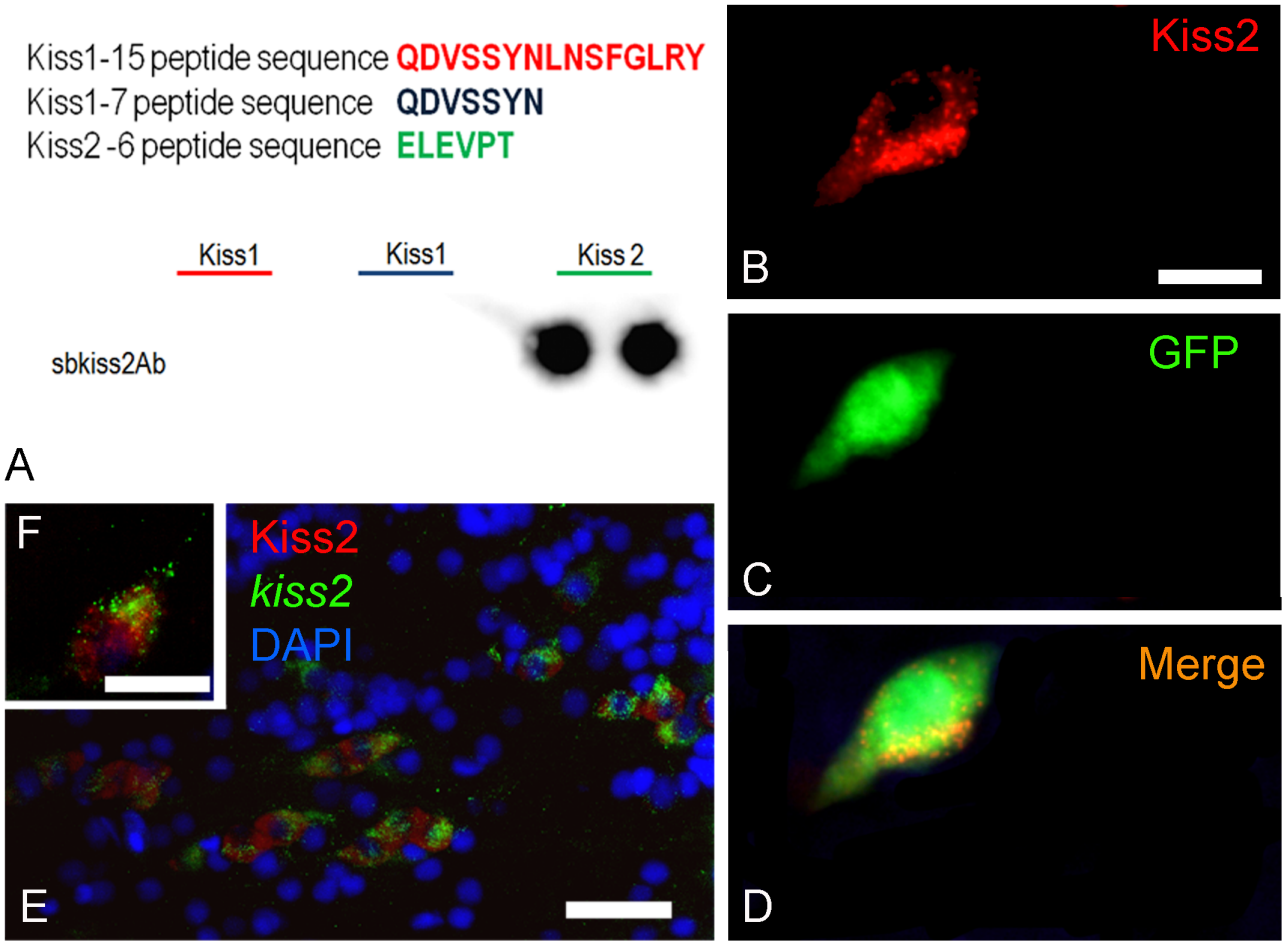
Specificity of the preproKiss2 antibody. (A) Immunoblotting demonstrating that the antibody directed against the C-terminus of the preproKiss2 recognizes the peptide ELEVPT sequence and does not cross-react with the preproKiss1 derived sequences. (B–D) Cells co-transfected with pCDNA3-Kiss2 (red in B) and pCDNA3-GFP (green) expression vectors exhibit immunoreactivity to the preproKiss2 antibody. Controls transfected with the empty vector are negative. Bar = 15 µm. (E–F) Cells labelled by the preproKiss2 probes (green) using in situ hybridization also exhibit immunoreactivity for preproKiss2 (red). E: Bar = 25 µm; F: Bar = 10 µm.

To produce antibodies against Kiss1 and Kiss2, the same strategy that proved successful in zebrafish [18] was used in sea bass. Antibodies were generated against the the N-terminal sequence of the mature peptide Kiss1 1–15 and C-terminal sequence of preproKiss2. However, due to the low titre of the Kiss1 antiserum, only the preproKiss2 could be used efficiently. Its specificity was verified by three different methods. First, Figure 1 A shows an immunoblot evidencing that the antibody recognizes the peptide to which it was designed. Second, cells transfected with a pcDNA3-kiss2 expression vector exhibit strong reactivity to the corresponding antibody (Figures 1B–D). Finally, cells labelled by the preprokiss2 probes using ISH also exhibit immunoreactivity for preproKiss2. Altogether, these data validate the use of the preproKiss2 antiserum for the detection of preproKiss2-expressing cells (Figures 1E–F).

#### Distribution of the Kiss2-expressing cells in the brain of the European sea bass

**Figure 2:**
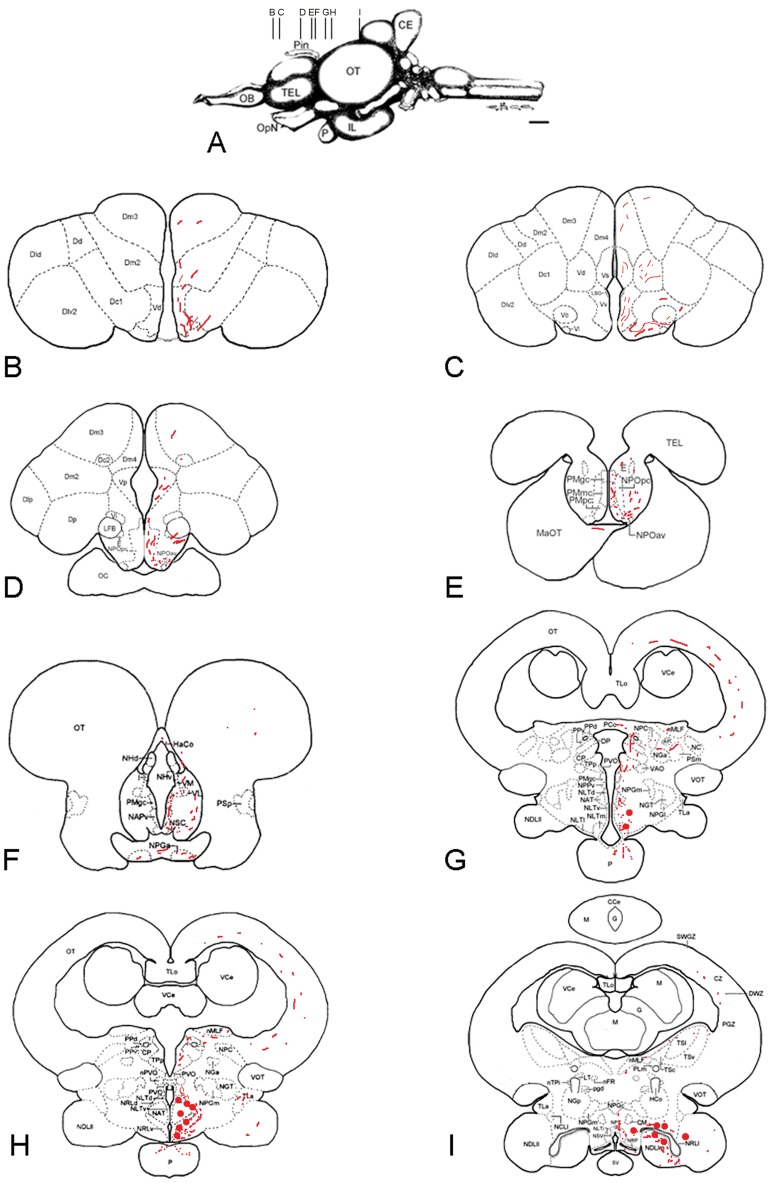
Distribution of preproKiss2-immunoreactive structures in the brain of the sea bass. (B,I) Shematic representation of preproKiss2-immunoreactive structures on representative transverse sections taken from the *Dicentrarchus labrax* brain atlas (Cerda-Reverter et al., 2001a, b; 2008). The level of these sections is shown in panel A taken from the atlas. Red circles represent the cell bodies and red dots the main fiber tracts. CCe corpus of the cerebellum; CE cerebellum; CP central posterior thalamic nucleus; Dlp lateral posterior part of the dorsal telencephalic area; Dm2 subdivision 2 of the medial dorsal telencephalic area; Dm3 subdivision 3 of the medial dorsal telencephalic area; Dp posterior portion of the dorsal telencephalon; E entopeduncular nucleus; HaCo habenular commissure; I intermediate thalamic nucleus; IL inferior lobe of the hypothalamus; MaOT marginal optic tract; NAPv anterior periventricular nucleus; NAT anterior tuberal nucleus; NC cortical nucleus; NDLIl lateral part of the diffuse nucleus; NDLIm medial part of the diffuse nucleus of the inferior lobe; NGT tertiary gustatory nucleus; NHd dorsal habenular nucleus; NHv ventral habenular nucleus; NLTI inferior part of the lateral tuberal nucleus; NLTm medial part of the lateral tuberal nucleus; NLTv ventral part of the lateral tuberal nucleus; nMLF nucleus of the medial longitudinal fasciculus; NPC central pretectal nucleus; NPGa anterior preglomerular nucleus; NPGc commissural preglomerural nucleus; NPGm medial preglomerural nucleus; NPOav anteroventral part of the parvocelullar preoptic nucleus; NPOpc parvocellular part of paraventricular organ; NPPv posterior periventricular nucleus; NPT posterior tuberal nucleus; NRLd dorsal part of the nucleus of the lateral recess; NRLl lateral part of the nucleus of the lateral recces; NRLv ventral part of the nucleus of the lateral recess; NRP nucleus of the posterior recces; NSC suprachiasmatic nucleus; LFB lateral forebrain bundle; OB olfactory bulbs; OC optic chiasm; OpN optic nerve; OT optic tectum; P pituitary; PCo posterior commissure; Pin pineal gland; PMgc gigantocellular part of the magnocellular preoptic nucleus; PMmc magnocellular part of the magnocellular preoptic nuycleus; PMpc parvocellular part of the magnocellular preoptic nucleus; PPd dorsal periventricular pretectal nucleus; PPv ventral periventricular pretectal nucleus; PSp parvocellular superficial pretectal nucleus; PVO paraventricular organ; SV saccus vasculosus; TEG tegmentum; TEL telencephalon; TLa nucleus of the torus lateralis; TLo torus longitudinalis; TPp periventricular nucleus of the posterior tuberculum; VCe valvula of the cerebellum; VL ventrolateral thalamic nucleus; VM ventromedial thalamic nucleus; VOT ventral optic tract; Vp postcommissural part of the ventral telencephalon. Scale bar = 1 mm.

**Figure 3:**
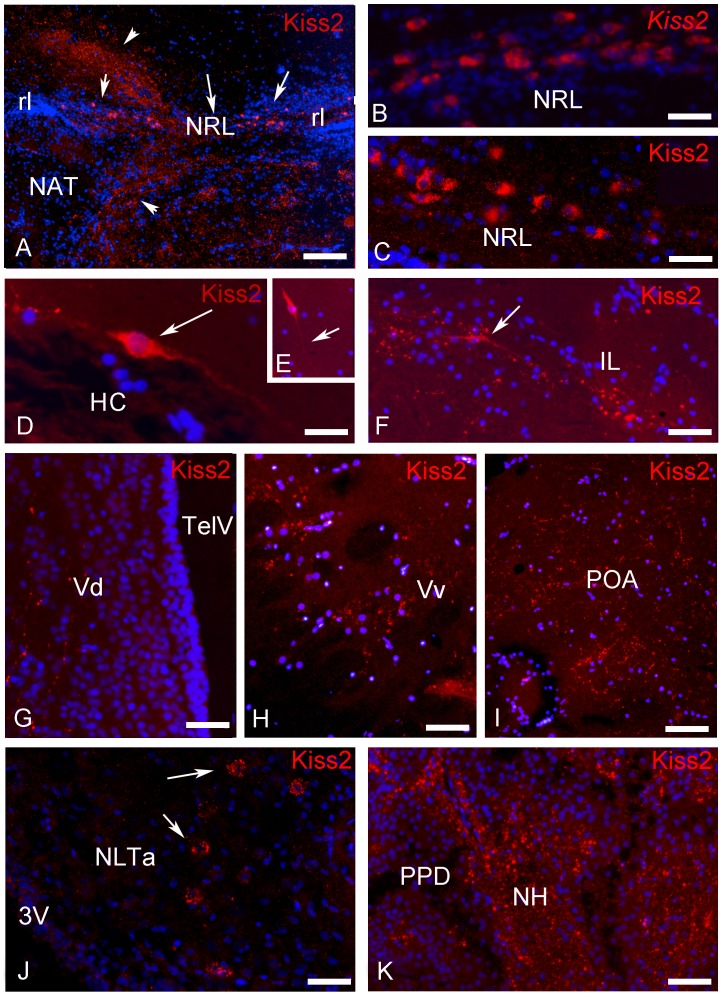
Transverse sections showing examples of preproKiss2-immunoreactive structures in the brain of European sea bass. (A) Low power view at the level of the hypothalamus showing positive cells in the region of the nucleus of the lateral recess (arrows). Please note the high density of immunoreactive-fibers heading towards the dorsal diencephalon and the ventromedial hypothalamus (arrowheads). Bar = 100 µm; (B–C) High power view at the level of the hypothalamus showing positive cells in the region of the nucleus of the lateral recess showing in (B) many *kiss2* mRNAs detected by ISH and in (C), the same area stained with the Kiss2 antibody. D: Bar = 30 µm; D: Bar = 25 µm. (D–F) Examples of isolated bipolar Kiss2-immunoreactive neurons (D) adjacent to the horizontal commissure (HC), (E) above the caudal part of the lateral recess and (F) in the inferior lobe (IL). D: Bar = 10 µm; E: Bar = 40 µm; F: Bar = 40 µm. (G–I) Immunoreactive fibers in the dorsal (G; Vv) and ventral (H; Vv) portions of the subpallium and lateral to the preoptic area (I; POA). G: Bar = 30 µm; H: Bar = 40 µm; D: Bar = 75 µm**.** (J) Examples of cells receiving immunoreactive innervation in the anterior nucleus lateralis tuberis (aNLT). The cells are completely surrounded by immunoreactive profiles (arrows). (K) Positive fibers in the neurohypophysis (NH) at the level of the proximal pars distalis (PPD). D: Bar = 30 µm.

In agreement with our previous data based on ISH [20], Kiss2-expressing neurons could be detected in the caudal hypothalamus with a very large distribution starting at the level of the horizontal commissure and extending to the caudal extent of the lateral recess (Figures 2 and 3). Also confirming previous data, the most immunoreactive neurons were detected consistently above the lateral recess of the third ventricle (Figures 2G–I, 3A and 3C). Figures 3B and 3C show these cell groups underlining the lateral recess and stained by ISH and IHC, respectively. The most anterior cells are detected on transverse section in the anterior hypothalamus around the horizontal commissure (Figures 2G and 3D), while the most caudal were seen scattered in the inferior lobe, not far but not necessarily in, the nucleus of the lateral recess (Figures 2I and 3E). In contrast to those of the lateral recess that were often round, these isolated cells often exhibited a bipolar shape (Figures 3D–F). Furthermore, additional less-immunoreactive cells could be observed in the mediobasal hypothalamus, just above the pituitary stalk (Figures 2G and 3J).

It was not possible to really trace the fiber tracts emerging from these cell bodies. However, it seemed that the majority of these fibers could in fact originate from this population. Indeed, the highest density of immunoreactive fibers was consistently detected in the hypothalamus. From the area encompassing the cell bodies, it seems quite clear that fibers ascend (Figures 2G–I and 3A) along the third ventricle within the thalamic region to innervate the dorsal and lateral thalamus. Immunoreactive fibers were also seen decussating within the posterior commissure (Figure 2G). Fibers also ascend in the mesencephalon and innervate the torus semicircularis and the optic tectum (Figures 2F–H).

From the positive cell bodies observed above the lateral recess a large contingent of fibers runs laterally and arches around the nucleus of the anterior tuberis to finally innervate the mediobasal hypothalamus (Figure 2H and 3A). At the level of the anterior portion of the tuberal region, fibers were frequently observed surrounding negative cells (Figure 3J). Many fibers also run more posteriorly towards the caudal hypothalamus and the regions surrounding the lateral recess and the posterior recess. Finally, the most caudal kiss2 projections were detected in the medial part of the caudal hypothalamus above the saccus vasculosus. In addition, conspicuous kiss2 fibers were also present in the inferior lobes of the hypothalamus (Figures 2H–I), especially in the medial portion of the diffuse nucleus of the inferior lobe.

Probably also from the cell bodies located in the dorsal hypothalamus, fiber tracts run anteriorly near the horizontal commissure and reach the caudal preoptic region (Figure 3I). Some course along the ventricle within the anterior periventricular nucleus and the magnocellular nucleus, while another portion runs more laterally along the optic tract and enter the pretectal region and the optic tectum. Some fibers where observed within the habenular commissure (Figure 2F). The most rostral Kiss2 fibers were observed in the anterior subpallial (Figures 2A–C and 3G–H) and pallial areas of the telencephalon. Fibers exhibiting very fine varicosities were particularly abundant in the dorsal extent of the subpallium. Such fibers were observed at all levels of the telencephalon up to the post-commissural nucleus, which marks the telencephalic-diencephalic transition (Figures 2 and 3). Additionally, in more lateral regions of the ventral telencephalon (Figure 3A–B), densely packed fibers were consistently observed running laterally (Figures 2 and 3). At the post-commissural level fibers probably coming from the preoptic area underlined the frontier of the lateral forebrain bundle, and enter the entopenduncular nucleus (Figure 2D). These fibers most likely originate from the more caudal parts of the brain since we could not detect any cell bodies in the olfactory bulb or telencephalon.

Finally, a prominent Kiss2 innervation was observed in the neurohypophysis at the level of the proximal pars distalis (Figure 2G–H and 3K).

#### Distribution of kiss receptors expressing cells in the brain and pituitary gland of the European sea bass

**Figure 4:**
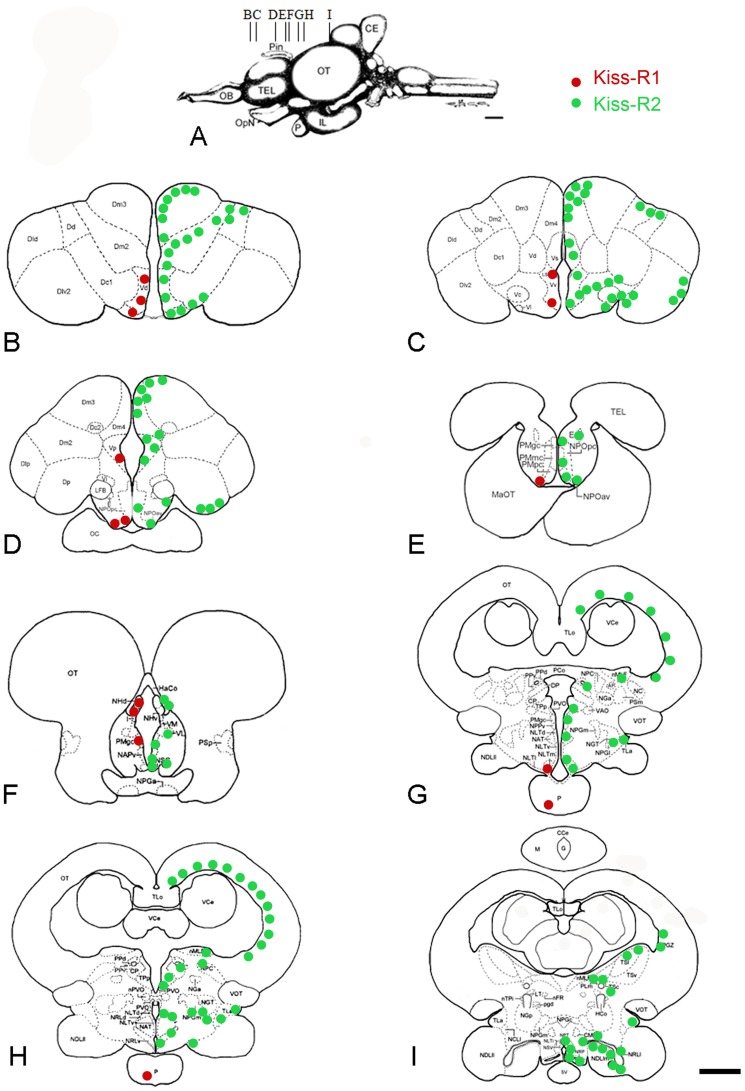
Distribution of *kiss-R1* and *kiss-R2 mRNAs* in the brain of the sea bass. Schematic representation of *kiss-R1* (red) and *kiss-R2* (green) expressing cells on representative transverse sections (B–I) taken from the *Dicentrarchus labrax* brain atlas (Cerda-Reverter et al., 2001a, b; 2008). The level of these sections is shown in panel A taken from the atlas. See list of abbreviations. Bar = 1 mm.

The distribution of *kiss-R1* and *kiss-R2* was investigated using fluorescent *in situ* hybridization in the brain and pituitary gland of male and female sea bass. No obvious sexual dimorphism was observed at the histological level. The data are summarized on Figure 4.

### Distribution of Kiss-R1 mRNAs

**Figure 5:**
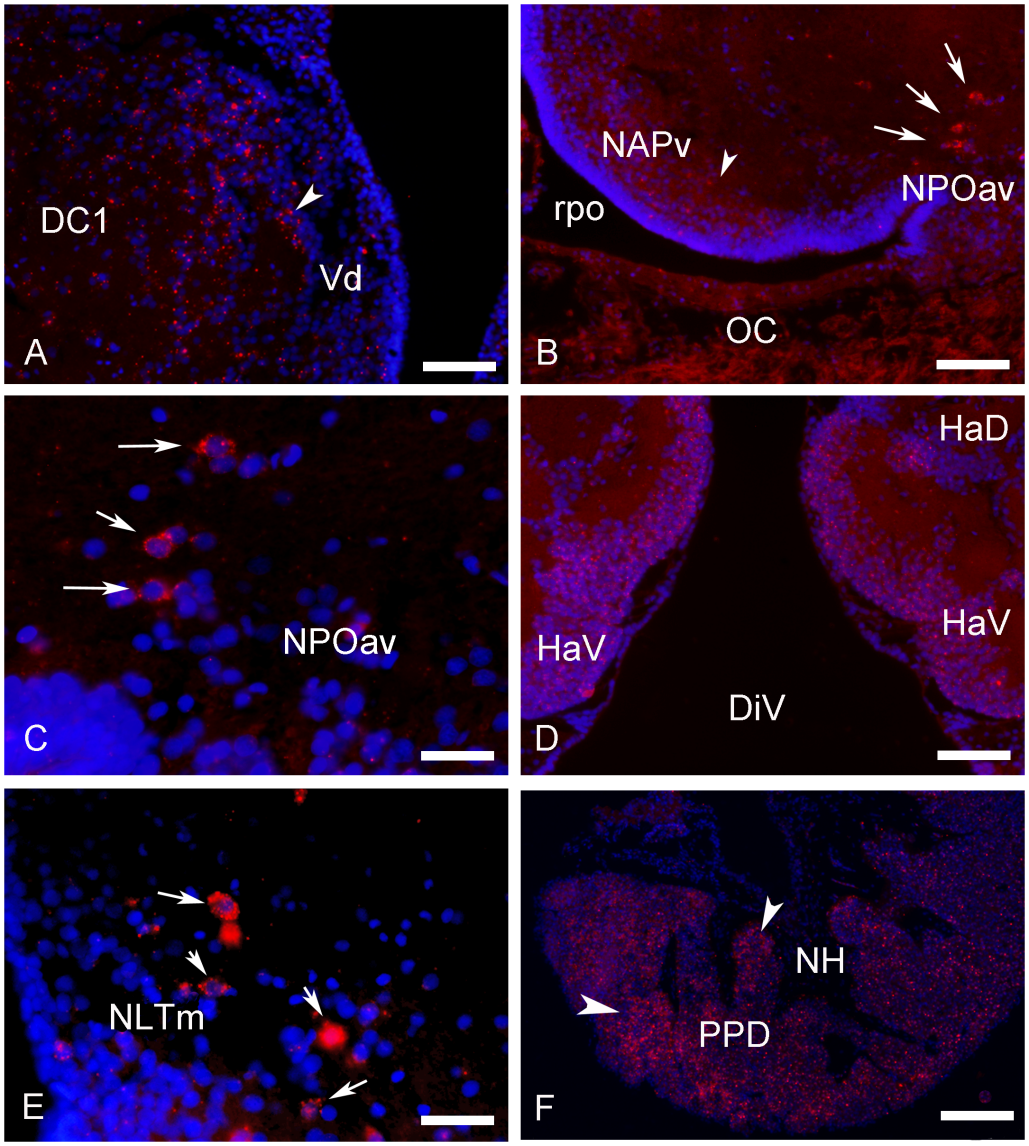
Transverse sections showing expression of *kiss-R1* messengers-expressing cells in the brain and pituitary gland of sea bass. (A) Small *kissr1*-positive cells in the ventrodorsal (Vd) part of the telencephalon. Bar = 30 µm. (B–C) *kiss-R1* mRNA expression signal in the parvocellular preoptic nucleus (pars anteroventralis, NPOav) at low (B; Bar = 75 µm) and high magnification (C; Bar = 15 µm). (D) In the habenular region numerous *kiss-R1* expressing cells are identified especially in the ventral habenula (Hav). Bar = 50 µm. (E) Into the mediobasal hypothalamus, the medial part of the nucleus lateralis tubersi (NLTm) presents small clusters of *kiss-R1* mRNA expressing cells, just above the pituitary stalk. Bar = 25 µm. (F) *Kiss-R1* mRNA (arrows) are detected in the pituitary gland of sea bass, especially at the level of the proximal pars distalis (PPD). Scale bar = 100 µm.

In both sexes, small cells expressing *kiss-R1* were detected in the subpallium of sea bass, notably in the ventral, dorsal and postcommissural extents (Figures 4B–D, 5A–B).

In the preoptic area, cells expressing *kiss-R1* mRNA were found in the anteroventral and ventral components of the parvicellular preoptic nucleus (Figures 4D–E, 5B–C). To a lesser extent few weakly-labeled positive cells were observed into the magnocellular preopticus nucleus (Figure 4F). A high expression of *kiss-R1* mRNA was detected in the habenula, in its ventromedial portion (Figures 4F and 5D). Very few round cells expressing *kiss-R1* are observed at the level of the mediobasal hypothalamus, in the medial part of the lateral tuber nucleus (Figures 4G and 5E). Finally, the pituitary gland of sea bass, and in particular the proximal pars distalis represented a site of high expression of *kiss-R1* mRNA (Figures 4G–H and 5F).

### Distribution of Kiss-R2 mRNAs

**Figure 6:**
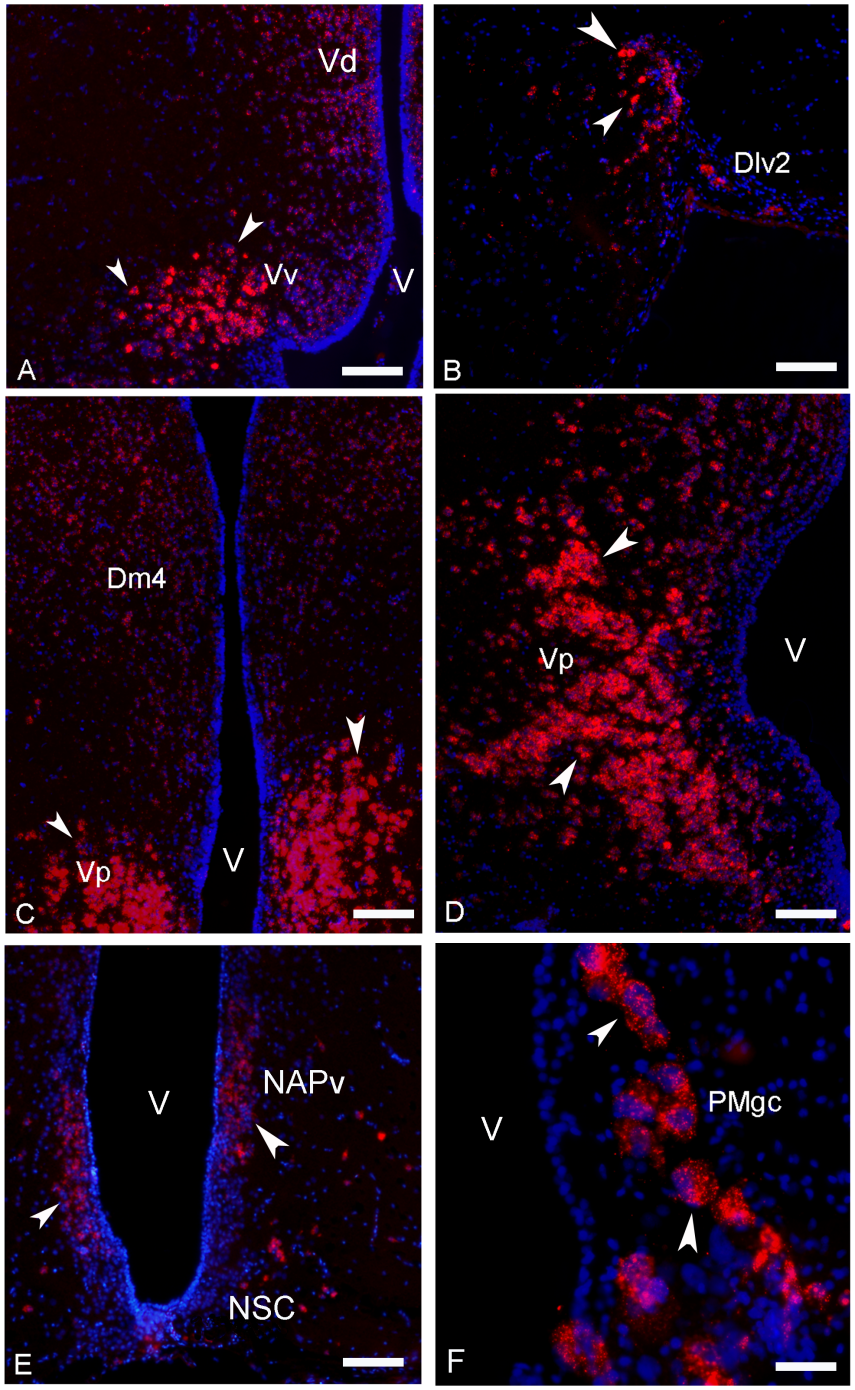
Transverse sections showing expression of *kiss-R2* messengers-expressing cells in the telencephalon, the preoptic area and the pituitary gland of sea bass. (A–D) A high expression for *kiss-R2* mRNAs was detected in the telencephalon, notably in the ventral (Vv in A), post-commissural (Vp in D) parts of the ventral telencephalon and in the dorsal area (Dlv2 in B). However, lower but significant expression of *kiss-R2* mRNAs was also observed into the medial dorsal (Dm4 in C) and in the supracommissural parts (Vs). A–E: Bar = 75 µm. (E–F) The preoptic area also contained many cells expressing *kiss-R2* messengers notably in the anterior (NAPv in E) and posterior portions of the periventricular preoptic nucleus. Expression was also consistently found in the magnocellular and gigantocellular portions (PMgc in F). In E: Bar = 40 µm.

In contrast to the discrete expression of *kiss-R1*, the brain of sea bass shows a very remarkable widespread expression of *kiss-R2* mRNA into the fore-, mid- and hindbrain. Starting from the telencephalon intensely stained *kiss-R2* cells were observed in the ventral area, mostly into the ventral, dorsal and post-commissural parts (Figures 4B–D, 6A,C–D). Other telencephalic nuclei showing *kiss-R2* expression include several subdivisions of the medial dorsal area (Dm2, Dm3, Dm4), the central dorsal area (Dc1), the ventrolateral dorsal area (notably the subdivision 2, Dlv2) and the posterior dorsal area (Figures 4B and 6B).

*Kiss-R2* expression was also prominent in the preoptic region, where all levels of the parvicellular nucleus contained many positive cells, as well as the different levels of the magnocellular preoptic nucleus (Figures 4D–F and 6E–F). Impressive kiss-R2 expression was also obvious in the giant cells of the gigantocellular part, as shown in Figure 6F. The periventricular anterior nucleus (Figure 4F), the suprachiasmatic nucleus and the most posterior nucleus of the preoptic region, the periventricular posterior nucleus (Figures 4G–H), contained few kiss2 mRNA expression sites. Weak kiss-R2 expression signal was detectable at the level of the entopenduncular nucleus (Figure 4E).

No *kiss-R2* expressing cells were observed in the epithalamic habenular nuclei of sea bass. Nevertheless, just lateral to this region, close to the habenular commissure, some positive cells have been identified (Figure 4F). In a more ventral location, many small kiss-R2 mRNA containing cells were revealed into the ventrolateral thalamus, the periventricular pretectal nucleus central nucleus of the pretectum (Figure 4F).

**Figure 7:**
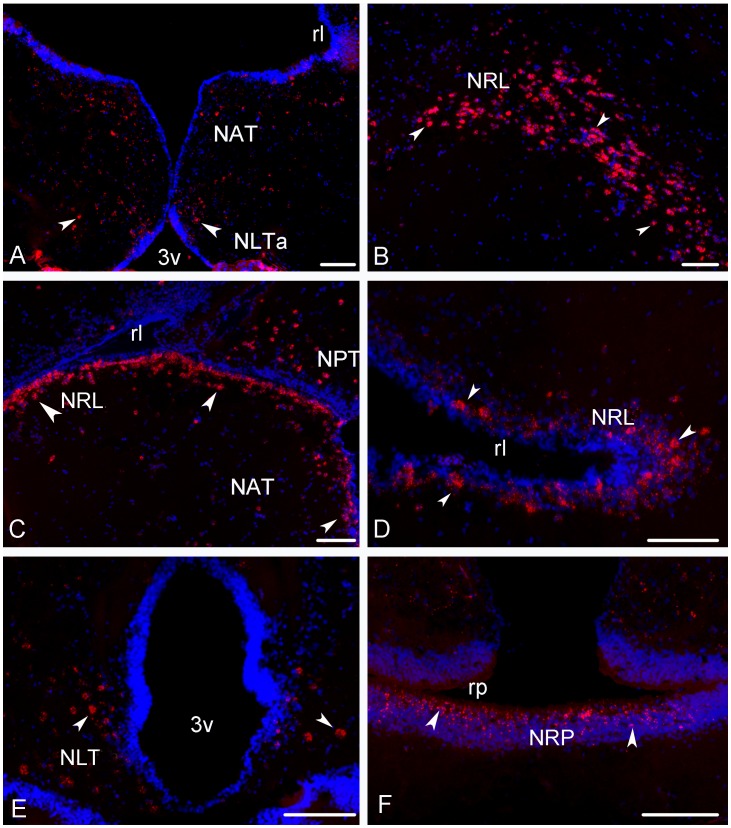
Transverse sections showing expression of *kiss-R2* messengers-expressing cells in the hypothalamus of sea bass. (A) *Kiss-R2* messengers expressing cells (arrowheads) in the nucleus anterioris tuberis (NAT) and the anterior nucleus lateralis tuberis (NLTa). Bar = 100 µm. (B–D) Strong expression of the *kiss-R2* mRNAs at different levels of the nucleus of the lateral recess (NRL) surrounding the lateral recess (rl). B: Bar = 75 µm; C: Bar = 75 µm; D Bar = 75 µm. (E–F) *Kiss-R2* messengers expressing cells (arrowheads) in the caudal nucleus lateralis tuberis (NLT) and in the nucleus of the posterior recess (NRP) surrounding the posterior recess (rp). E: Bar = 75 µm; F: Bar = 100µm.

Dispersed positive cells were found in the nucleus lateralis tuberis from its anterior (Figures 4G–H and 7A) to more caudal parts (Figure 7E). A very prominent population of *kiss-R2* expressing cells was identified in the hypothalamus. Since the very beginning down to the caudal extent of the lateral recess numerous *kiss-R2* mRNA expressing cells were consistently observed (Figures 4G–H and 7B–D). However, while Kiss2-positive cells were in majority located above the lateral recess, *kiss-R2*-expressing cells were preferentially found below the lateral recess (Figure 7C). These cells are present all along the lateral recess including in the inferior lobe, where they appeared to surround the recess (Figure 7D). In the medial part of the lateral tuber (NLTm) *kiss-R2* expressing cells are located just above the pituitary gland (Figures 4G and 7E). The inferior margin of the posterior recess showed many moderately stained *kiss-R2* cells (Figures 4I and 7F).

More posteriorly, isolated positive cells were observed in the nucleus of the medial longitudinal fascicle (Figures 4H–I) and at different levels of the midbrain and torus semicircularis. In the periventricular grey zone and the deep white zone of the optic tectum, small *kiss-R2* mRNA containing cells are consistently detected (Figures 4G–I).

#### GnRH1 neurons do not seem to express kiss receptors

**Figure 8:**
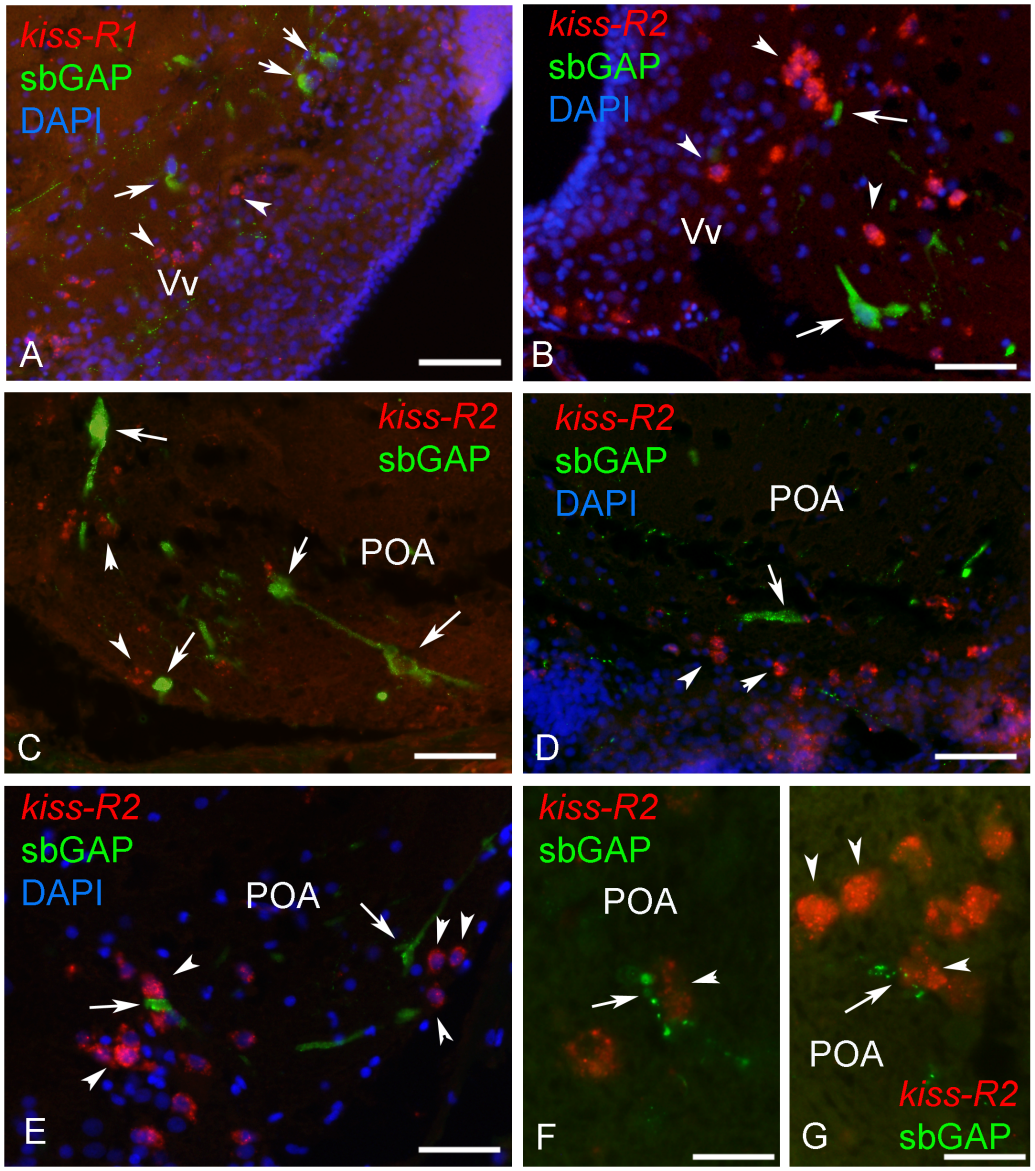
*Kiss-R2* mRNAs are not expressed in GnRH1 neurons in the brain of sea bass. Relationships between *kiss-R1* (A) or *kiss-R2* (B–G) expressing cells (red) and sbGAP (GnRH1)-immunoreactive neurons (green) in the ventral telencephalon (Vv) and the lateral preoptic area (POA). While kiss-R expressing cells (arrowheads) are often found in close vicinity of GnRH1-expressing cells (arrows), no co-expression could be detected. Occasionally, GnRH1 immunoreactive dendrites or varicose fibers were in close apposition to kiss-R2 expressing cells (B,E–G). A: Bar = 50 µm; B: Bar = 30 µm; C: Bar = 30 µm; Bar = 50 µm; D: Bar = 40 µm; E; Bar = 40 µm; F: Bar = 25 µm; G: Bar = 25 µm.

A particular attention was given to the potential expression of kiss receptors in the GnRH neurons, focusing in particular on the ventral telencephalon and the ventral preoptic region where most GnRH1 expressing neurons are located in the European sea bass [26]. An antibody against the GAP fragment of the preproGnRH1 (sbGnRH) was used for coupling with *kiss-R1* and *kiss-R2* ISH. This antibody was previously shown to be highly specific for staining sbGnRH (GnRH1) neurons in sea bass [27], [28]. Figure 8 shows that, although *kiss-R1* (Figure 8A) and *kiss-R2* (Figures 8B–E) expressing cells in the ventral telencephalon and ventral preoptic area, are often found in close proximity to GnRH1 neurons, we could not detect a single case of co-expression. We found some examples of GnRH1-immunoreactive fibers contacting *kiss-R2* expressing cells (Figures 8F–G) or examples of GnRH neurons located very close to *kiss-R2* expressing cells (Figure 8E).

#### Kiss-R2 mRNAs are present in a variety of neuronal systems

**Figure 9:**
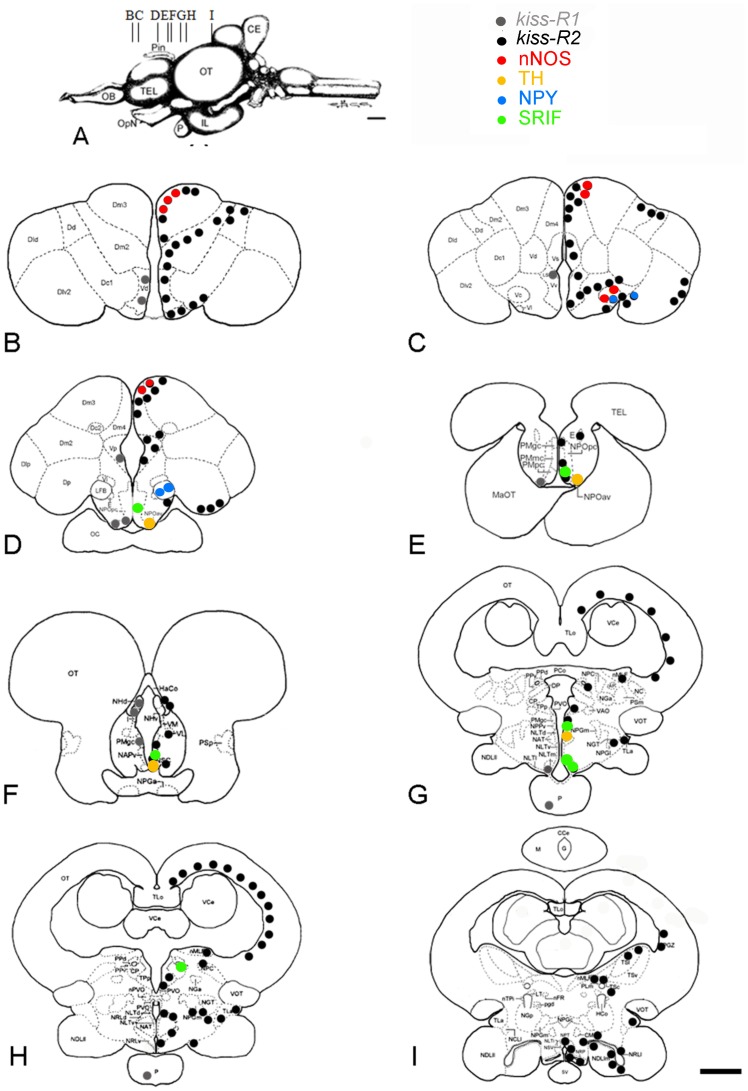
Distribution of *kiss-R2* mRNAs in phenotypically-identified neurons in the brain of sea bass. Shematic representation of phenotypically identified kiss-R2 (black) expressing cells on representative transverse sections (B–I) taken from the *Dicentrarchus labrax* brain atlas [21]–[23]. The level of these sections is shown in panel A taken from the atlas. For abbreviations, see Figure 2. Bar = 1 mm.

**Figure 10:**
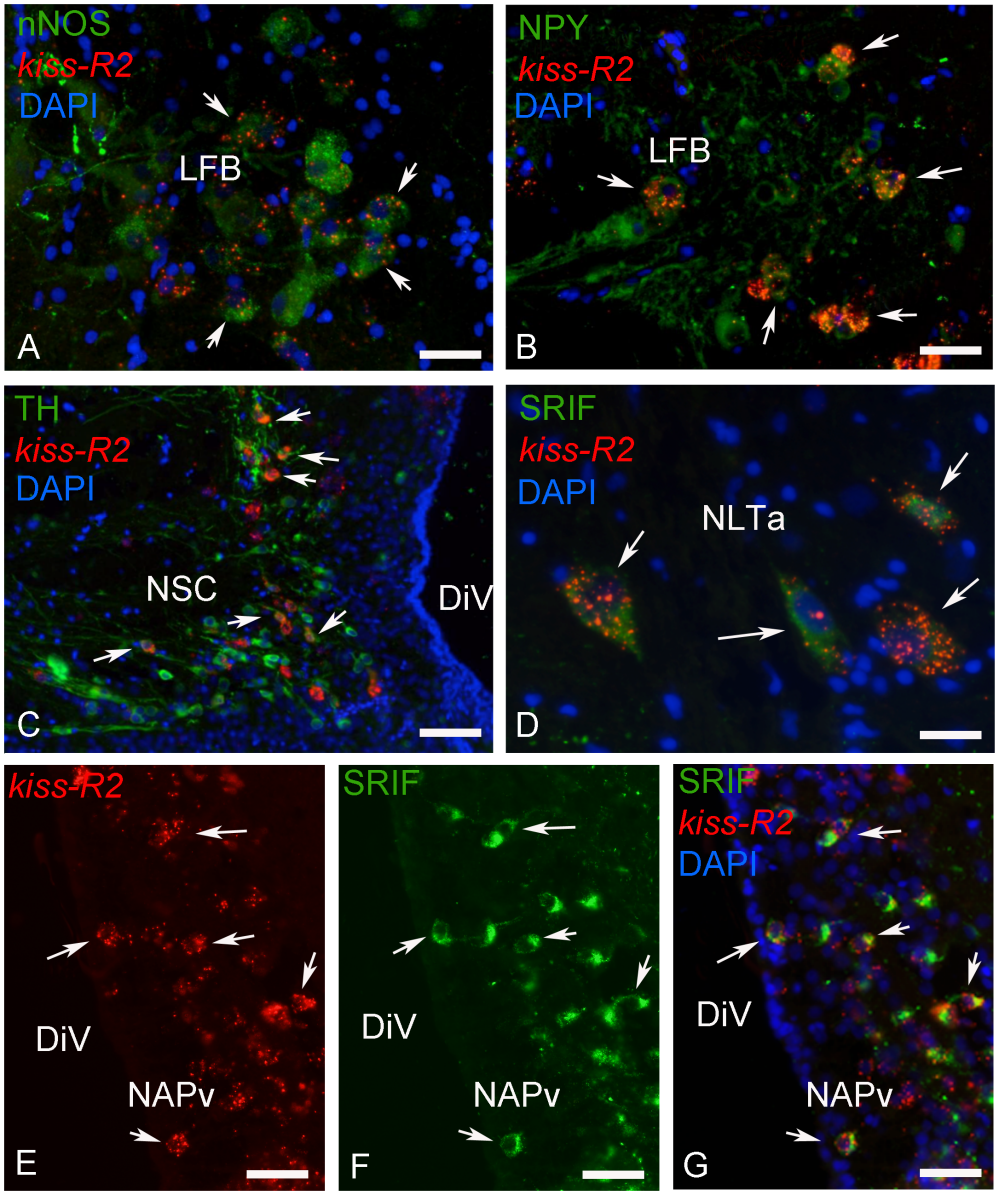
Expression of *kiss-R2* mRNAs in chemically identified neurons in the brain of sea bass. Examples of *kiss-R2*-expressing cells exhibiting immunoreactivity to nNOS (neuronal nitric oxide synthase in A), NPY (neuropeptide Y in B), TH (tyrosine hydroxylase in C) and SRIF (somatostatin in D–G). A: Bar = 10 µm; B: Bar = 10 µm; C: Bar = 20 µm; D: Bar = 8 µm; D: Bar = 40 µm; E-G: Bar = 15 µm.

In order to start identifying the phenotype of the cells expressing kiss receptors, we performed combined in situ hybridization and immunohistochemistry for neuronal nitric oxyde synthase (nNOS), tyrosine hydroxylase (TH), neuropeptide Y (NPY) and somatostatin (SRIF). We found many examples of neurons expressing *kiss-2R* messengers and nNOS, in particular in the dorsal telencephalon and around the lateral forebrain bundle (Figures 9B–D and 10A). In this latter region, many but not all NPY-positive cells also exhibited expression of kiss-R2 messengers (Figures 9C–D and 10B). Co-expression was also evident in part of the TH-positive cells of the anterior ventral preoptic area or in the suprachiasmatic nucleus (Figures 9D–F and 10C).

Very interestingly, we observed in two different males that 100% of the neurons immunoreactive for somatostatin expressed kiss-R2 (Figures 9D–H). This is true for the numerous cells present at the different levels of the periventricular preoptic nucleus (Figures 10E–G), but also for the cells observed in the thalamic area and the mediobasal hypothalamus (Figure 10D).

## Discussion

### Distribution of Kiss2 Immunoreactive Structures

Because of the similarity between Kiss1 and Kiss2 peptides, making it difficult to raise specific antibodies, we used a strategy similar to that already employed successfully to generate antibodies against kisspeptins in zebrafish [18]. Unfortunately, while this strategy worked well in the case of preproKiss2, we failed to obtain high titer antibodies for preproKiss1. In the case of preproKiss2, the different tests of specificity that were carried out demonstrated that such an antibody can be used to detect Kiss2-expressing cells in the brain of sea bass. Despite the fact that IHC performed on the same slide after ISH results in the loss of part of the hybridization signal, we could evidence that *kiss2* messengers and proteins coexist in the same cells of the hypothalamus. Immunohistochemistry also confirmed the presence of positive cells in a large region of the hypothalamus, encompassing the mediobasal, dorsal and caudal regions. As already observed with ISH [20], we found that the most numerous and immunoreactive cells were those located above and around the lateral recess of the hypothalamus. This population extends, under the form of scattered cells, anteriorly up to the horizontal commissure and caudally to the inferior lobe of the hypothalamus. Interestingly, the size and shape of the neurons observed in the very anterior or posterior parts of the hypothalamus, were different from those of the main population observed in the nucleus of the lateral recess. These neurons were usually isolated, bipolar and exhibited sometimes very long dendrites or axonal cones. This suggests that such neurons could in fact represent a population different from that of the dorsal nucleus of the lateral recess. In contrast with our previous study, we did not detect any positive cell bodies in the preoptic area, which in fact could be due to the scattered character of such cells.

The interest of immunohistochemistry above in situ hybridization is its capacity to reveal the organization of the projections of cell bodies. In the present study, positive varicose fibers were detected in the ventral telencephalon and diencephalon with a distribution very similar to that observed in zebrafish [18]. Such fibers were abundant in the neuroendocrine areas, but also in extra-hypothalamic territories, such as the pallial and subpallial regions, the optic tectum and the torus semicircularis. This suggests that in addition to potential functions in the neuroendocrine regulation of pituitary activity, kissspeptins could act as neuromodulators of sensorial inputs.

### Distribution of Kiss-R1 and Kiss-R2 in the Brain of European Sea Bass

Surprisingly, there is little information on the distribution of kisspeptin receptors in the brain of mammals. Nevertheless, a study based on a transgenic Gpr54 LacZ knock-in mouse model indicated a very large distribution of GPR54 in the septum, rostral preoptic area, thalamus, posterior hypothalamus, periaqueductal grey, supramammillary and pontine nuclei, and dorsal cochlear nucleus [39].

In the European sea bass, while *kiss-R1* mRNAs exhibited a modest expression in limited regions, *kiss-R2* messengers presented a very large distribution and a high level of expression in some areas. Expression of the two receptors present a certain overlap in several regions, such as the ventral telencephalon or the preoptic area, but in most other nuclei, *kiss-2R* is by far the predominant receptor form. Additionally, it is important to record that most brain regions exhibiting *kiss-R2* receptors also exhibit Kiss2 fibers. This was particularly obvious in the pallium, the entopeduncular nucleus, the thalamic area, the optic tectum or the inferior lobe.

The overlap between *kiss-R2* receptors and Kiss2 fibers is very striking in at least two brain regions. The first one consists in the central telencephalon where an astonishing expression of *kiss-R2* was observed. The second one corresponds to the region surrounding the lateral recess of the hypopthalamus, which not only receives a heavy Kiss2 innervation, but also is a surrounded by a very high number of *kiss-R2* expressing cells. Thus, in all these regions it is very likely that Kiss2 is the biologically active ligand of these Kiss-R2 expressing cells.

The fact that kisspeptin receptors were also found in the optic tectum, the torus semicircularis or the tegmentum of the midbrain indicates that, similar to the mammalian situation, kissspeptins are likely to affect a large number of neuronal systems in fishes.

### GnRH Neurons do not Seem to Express Kiss Receptors

The GnRH system of the European sea bass has been extensively studied and it is actually the only teleost in which specific antibodies were developed against the GAP fragment of the preproGnRH precursors. Such studies clearly evidenced that sea bream GnRH neurons are the major contributors to the pituitary GnRH innervation. In this study, a careful investigation of the GnRH1 (sbGnRH) expressing cells of the ventral telencephalon and preoptic area was performed by coupling in situ hybridization of *kiss-R2* or *kiss-R1* with sbGAP immunohistochemistry. However, we could not observe any cell showing co-expression, which tends to indicate that GnRH neurons are not direct targets of kiss systems in the ventral telencephalon and the preoptic area. So far, attempts to perform double Kiss2/sbGnRH immunohistochemistry also failed to clearly show direct contact between the two systems. In zebrafish, detailed investigations equally failed to evidence kiss receptors in GnRH neurons and apart from some potential direct contacts between Kiss2 fibers and GnRH3 neurons, there is very little morphological evidence as for direct interactions [18]. Similarly, a recent study in medaka failed to detect any kiss receptor mRNA in GnRH1, GnRH3 or GnRH2 neurons of the medaka [40]. While early studies claimed that all three populations of tilapia GnRH neurons express *kiss* receptor mRNA based on single cell PCR [41], there is so far no morphological evidence in any fish that it is indeed the case. These data are thus in contrast with those obtained in mammals showing that KISS1 stimulates GnRH release through mostly postsynaptic effect, although a minor presynaptic component has been reported [39], [42], [43]. Recent data in mouse have pointed out that nNOS synthesizing neurons could be implicated in the transmission of the KISS1-mediated estrogenic positive feedback onto GnRH neurons [33].

### Searching for the Phenotype of Kiss Receptors Expressing Cells

In front of the lack of evidence suggesting interactions between kisspeptin and GnRH neurons in the brain of zebrafish, we decided to start investigating other neuronal systems potentially of interest: nNOS, TH, NPY and somatostatin. The choice for these particular candidates was dictated by the fact that all these factors are documented to influence gonadotrophin and/or growth hormone secretion in teleost fishes [44]. The present study also provides strong evidence that *Kiss-R2* are expressed in the magnocellular neurons of the preoptic nucleus, which is in agreement with a recent study in medaka showing that isotocin and vasotocin neurons express Kiss receptors [40].

Neuronal NOS (nNOS) belongs to a family of enzymes catalyzing the production of nitric oxide a gaseous neurotransmitter implicated in brain development and functioning [45]. In the mouse, there is accumulating evidence to show that NO is important in the regulation of reproduction, estrogen feedback [46], [47] and the regulation of GnRH neurons activity [33]. In fish, the expression of nNOS has been described in different species notably in the telencephalon and diencephalon [48], [49], in line with the present study. Additionally, recent data indicate a role for NO in the regulation of gonadotrophin release, but this effect is most likely due to expression of nNOS in the gonadotrophs [50]. The present data showing expression of kiss-R2 in NOS positive cells provide a first evidence for potential interactions that are currently under more accurate investigation.

Neuropeptide Y is a 36 amino-acid peptide largely expressed in the brain of teleosts in particular in the ventral and lateral telencephalon [51], [52]. This peptide is well known for its involvement in the neuroendocrine control of reproduction, growth and feeding behavior [53]–[55]. In the European sea bass, NPY was shown to induce LH secretion, however, this effect was dependent upon the energetic status [56]. NPY is thus a serious candidate for potential functions on growth/reproduction interactions in fishes and the finding of *kiss-R2* in NPY neurons in the lateral telencephalon is significant given that NPY is massively expressed in regions with high expression of *kiss-R2* such as the pallial regions [51]. Clearly, this awaits further detailed studies.

In the anterior preoptic area of fish, tyrosine hydroxylase neurons have been shown to be dopaminergic in nature [57]–[59]. Dopamine is a well-established regulator of gonadotrophin synthesis in many fish, but not in marine species [60]. In those fish where dopamine inhibits gonadotrophin release, the neurons responsible for this effect are located in the anteroventral preoptic region [58]. Such neurons also exist in the sea bass and part of them expressed *kiss-R2 mRNA* as shown in the present study. Although, the roles of these DA/*kiss-R2* expressing neurons are unknown, it is worth mentioning that such neurons were shown in other species to express estrogen receptors [61] and to interact with the GnRH systems [62], [63].

Somatostatin has been isolated in 1973 on the basis of its capacity to inhibit growth hormone release [64]. Somatostatin is a very conserved peptide that also acts as an inhibitor of growth hormone secretion in teleosts [65]–[70]. Using antibodies generated against somatostatin 1–14 [37], the distribution of somatostatin immunoreactive cells was reported for the first time in teleosts in the brain of the goldfish [38]. The data obtained in European sea bass with the same antibodies are extremely similar by showing the presence of positive cells in the anterior and posterior periventricular preoptic nucleus, the thalamic region and the anterior nucleus lateralis tuberis. A remarkable outcome of the present study was that all somatostatin positive neurons studied in two different males exhibited expression of *kiss-R2*. This result points to potential function of kisspeptins, and most likely Kiss2, in regulating SRIF neurons and thus growth hormone release.

In conclusion, this work further documents the expression of Kiss2 producing neurons in the brain of the European sea bass and examined the relationships with *kiss-R mRNA* expressing cells. Given the high correelation between Kiss2 fibers and *kiss-R2* expressing cells there is little doubt that, similar to the zebrafish, Kiss2 neurons will mostly act through Kiss-R2. An important observation of this work is the very large distribution of Kiss2 fibers and kiss-R2 mRNA expressing cells in the brain of the European sea bass. This indicates that Kiss2 is likely to play a much wider range of functions than previously thought. Strickingly, *kiss-R2* mRNAs could not be observed in any GnRH1 neurons while, in contrast, 100% of SRIF neurons in males expressed kiss-2R suggesting some roles of Kiss2 in the regulation of growth hormone, among other functions, notably directly at the pituitary level.
